# Medical and Literary Weekly

**Published:** 1859-07

**Authors:** 


### MEDICAL AND LITERARY WEEKLY.

Though we have, upon a former occasion, noticed favorably this new enterprise, we cannot forbear to call attention to it again.

It is especially medical men who should sustain this movement to enlighten the people in reference to matters that are not only of the first importance to the wellbeing of society, but which lie at the foundation of the appreciation upon the part of the public of the medical profession

We ar glad to learn that there is already a very considerable subscription list, and that it is steadily increasing; but its circulation might be doubled in a very short time, if the medical men of the country would take some interest in having it circulated in their respective communities. There is, perhaps, scarcely a respectable physici n anywhere, who could not induce a half dozen families to subscribe for this paper, and scarce y one of these would, in a short time, be willing to do without it.

We regard it as an exceedingly valuable paper, and if liberally sustained, will accomplish a great work. It is a weekly Journal, published in Atlanta, and devoted to literature and a diffusion among the people, of a knowledge of Physiological and Hygienic laws, and the exposure of the untold villainies practiced in the name of Medicine. Subscription price $2. Address Drs. Taliaferro & Toomas, Atlanta, Geo.

